# Effectiveness of Physical Activity-Led Workplace Health Promotion Interventions: A Systematic Review

**DOI:** 10.3390/healthcare13111292

**Published:** 2025-05-29

**Authors:** Shichao Zhang, Mingjian Nie, Jiale Peng, Hong Ren

**Affiliations:** Key Laboratory of the Ministry of Education of Exercise and Physical Fitness, Beijing Sport University, Beijing 100061, China; 2020110091@bsu.edu.cn (S.Z.); nie.mj@bsu.edu.cn (M.N.); peng@bsu.edu.cn (J.P.)

**Keywords:** workplace, health intervention, physical activity led, success rate of the interventions

## Abstract

**Background**: With increasing work pace and stress, sedentary office habits and insufficient physical activity (PA) pose significant threats to employee health and organizational productivity. Physical activity-led workplace health interventions (PAWHIs) have gained attention due to their multifaceted benefits for employees’ physical and mental health. This systematic review aims to evaluate the effectiveness of PAWHIs and examine the success rates of PA combined with various supplementary intervention approaches in improving employee health. **Methods**: This study was conducted according to the PRISMA 2020 statement guidelines. A systematic search was performed across four databases (PubMed, Web of Science, EBSCO, and Scopus) for randomized controlled trials (RCTs) published between 2013 and 2023. The Cochrane risk-of-bias tool was used to assess the risk of bias in the included studies. **Results**: After screening, 40 studies meeting the criteria were finally identified and subjected to quality assessment. The primary intervention measures of PAWHIs focused on education, physical activity, and dietary interventions. Fifty percent of the studies adopted multimodal combined intervention schemes involving two or more types of interventions. The most common intervention durations were 12 weeks (9 studies) and 24 weeks (24 studies). An analysis of the various intervention effects of PAWHIs revealed that the most successfully improved outcomes were increased physical activity levels (26/32), reduced psychological stress (4/5), and improved dietary habits (12/19), with over 60% of the related studies reporting positive effects. Additionally, improvements were also commonly observed in body composition (16/29) and clinical health outcomes (15/27). **Conclusions**: PAWHIs have positive effects on improving employee health status and promoting healthy behaviors, particularly in increasing PA levels and reducing psychological stress. However, interventions need to be contextualized and further optimized to achieve more comprehensive and sustainable health outcomes.

## 1. Introduction

With the increasing health awareness among enterprises and employees, the workplace has gradually become a key environment influencing employee health and well-being [1,2]. Accelerated work pace, poor work habits, sedentary behavior, overtime work, and other unhealthy lifestyles pose serious threats to employees’ physical and mental health [3,4,5]. These issues not only affect employees’ personal quality of life but also negatively impact corporate productivity and economic performance [6,7]. For instance, a 2022 employee health report from a Fortune 500 company in the IT industry revealed that the prevalence of metabolic syndrome among long-term sedentary programmers was as high as 32%, significantly exceeding the 15% rate in the general population. These health issues result in an average increase of 3.5 days of absence per employee per year, causing a direct loss of production efficiency of $2800 [6].

Current research indicates that the workplace accounts for up to 60% of employees’ waking hours [8], making it an internationally recognized setting for health promotion and disease prevention [9]. Workplace health intervention programs have garnered widespread attention due to their potential to enhance employees’ physical and mental well-being [10,11,12]. Among these, physical activity-led workplace health interventions (PAWHIs) have emerged as a popular approach. PAWHI refers to comprehensive health promotion programs implemented in workplace settings, with the core objectives of increasing physical activity (PA), fostering regular exercise habits, and ultimately improving employees’ physical and mental health and work performance. Numerous studies have demonstrated the effectiveness of PA in reducing chronic disease risks, improving body composition and physiological function, alleviating psychological stress and symptoms of depression and anxiety, and enhancing job satisfaction and work performance [13,14,15]. A meta-analysis specifically showed that increasing PA levels led to a 23% reduction in hypertension incidence, a 31% decrease in depression symptom scores, and a 14% improvement in work performance [12]. Additionally, a Fortune 500 company reported that through multifaceted interventions such as workplace exercises, sports clubs, and step counting via smart wristbands, employees’ average daily steps increased by 42%, waist circumference significantly decreased, and annual medical expenditures were reduced by 18% [16]. These findings corroborate the health-improving effects of PAWHIs, leading more enterprises to favor PAWHIs over traditional health intervention measures.

However, critical gaps remain in current PAWHI research, necessitating systematic reviews to address them. First, while the overall effectiveness of PA-led health interventions has been established, significant variations exist across studies in terms of intervention strategies (e.g., exercise type, intensity, duration), supplementary approaches (e.g., health education, psychological support, environmental modifications), and target populations. Further synthesis and summarization of the literature are required to guide the selection of appropriate intervention types and strategies that align with corporate objectives. Second, there is a lack of systematic analysis on the success rates of different PAWHI strategies. Research has indicated that standalone exercise interventions yield limited improvements in physical and mental health, whereas integrated approaches combining exercise with cognitive and technical enhancements demonstrate superior outcomes [17].

Therefore, this study aims to systematically review the relevant workplace health intervention literature, focusing on empirical PAWHI research. By analyzing intervention characteristics—including methods, evaluation metrics, and outcomes—we seek to elucidate the features of PAWHI and assess the success rates of various supplementary intervention approaches and combinations. The findings will provide a scientific basis for designing and implementing workplace health intervention programs while offering practical guidance and recommendations for enterprises and health managers.

## 2. Materials and Methods

This systematic review adheres to the PRISMA 2020 guidelines (Preferred Reporting Items for Systematic Reviews and Meta-Analyses), a standardized protocol comprising 27 items grouped into 7 domains. This system evaluation has submitted a registration application on the PROSPERO platform and is currently under review. The registration submission number is PROSPERO 2025 CRD420251047695. The final registration number will be updated through the Supplementary Materials or the officially published version. The primary research objectives and specific study questions are detailed in Supplementary Material S1.

### 2.1. Literature Search

To comprehensively capture diverse studies in this field, a systematic literature search was conducted across four major academic databases: PubMed, Web of Science, EBSCO, and Scopus. The search covered publications from January 2013 to December 2023, ensuring broad temporal coverage of relevant research. The search terms included the following: “Physical Activity”, “Occupational Group”, and ”Workplace”. The search strategy combined subject headings (MeSH terms) and free-text keywords, with the specific details presented in Table 1. Boolean operators (“AND” and “OR”) were applied to construct precise search queries. Search strategy: #1 AND #2 AND #3.

### 2.2. Eligibility Criteria

This systematic review examines PAWHI. The analysis evaluates intervention effectiveness across multiple health domains, including the following: physical activity levels, dietary and nutritional status, psychological stress, body composition, and disease-related health outcomes. A narrative summary analysis of intervention success rates was performed [18]. This study established literature selection criteria following the PICOS framework (Participants, Interventions, Comparisons, Outcomes, Study Design), with specific inclusion/exclusion criteria detailed in Table 2.

### 2.3. Data Extraction

Two authors (S.C. and M.N.) independently screened the literature following a standardized procedure. First, potential studies were evaluated based on their titles and abstracts to identify those relevant to the research question and meeting the predefined inclusion criteria. Duplicate records and studies deemed irrelevant after abstract or full-text review were excluded. Finally, the selected studies underwent full-text analysis to determine their eligibility for final inclusion. Any discrepancies between the researchers were resolved through discussion until consensus was reached.

The entire screening process was documented in a flowchart following the PRISMA guidelines. For data extraction, team members worked independently, compiling the key characteristics of the selected articles into a structured table containing the following information: author/year, country, occupation/company type, study objective, intervention duration, intervention type, and outcome type. This tabulated data facilitated the systematic understanding and interpretation of the included studies. The specific content can be found in Table 3.

The intervention types are classified into Educational Interventions; Physical Activity Interventions; Dietary Interventions; Other Intervention Types. The outcome types are classified as Clinical Health Outcomes, Body Composition, Diet and Nutrition, Physical Activity and Function, Sleep Habits and Quality, Psychological Health Outcomes (e.g., Depression, Anxiety, Self-Efficacy, Stress), and Work-Related or Organizational Outcomes.

### 2.4. Success Rate Definition

Based on the significant improvements observed across studies, this research defined the “success rate” as follows: the proportion of studies that demonstrated statistically significant improvements (*p* < 0.05) or clinically relevant improvements in at least one primary outcome measure when comparing the intervention group with the control group, either post-intervention or during follow-up [19]. The specific criteria were as follows:

(1) Statistical significance: determined based on the *p*-values reported in each study.
(2) Clinical relevance: for clinical health indicators (e.g., blood pressure and blood glucose), the degree of improvement had to meet the minimal clinically important difference (MCID) recommended by clinical guidelines.
(3) Handling of mixed results: if some indicators within a given health domain showed improvement while others remained unchanged in the same study, it was still considered a partial success for that domain.

### 2.5. Risk-of-Bias Assessment

The Cochrane risk-of-bias tool is widely recognized as the gold standard methodology for evaluating the quality of RCTs. In this study, all included RCTs were systematically assessed using the Cochrane risk-of-bias criteria, which examines seven key domains: random sequence generation, allocation concealment, blinding of participants and researchers, blinding of outcome assessment, completeness of outcome data, selective reporting, and other potential sources of bias. In accordance with the Cochrane risk-of-bias tool, two authors classified each study as “high risk”, “unclear risk”, or “low risk”. Specific evaluation criteria are provided in Supplementary Material S2. Based on these criteria, study quality was categorized into three levels: studies meeting five or more criteria were classified as low risk of bias, those meeting three to four criteria were classified as moderate risk of bias, and those meeting fewer than three criteria were classified as high risk of bias [20]. The results of the risk-of-bias assessments were graphically represented using the Review Manager 5.4 software.

**Table 3:** Characteristics of the selected studies.

Author/Year	N	Occupation or Company Type	Objective	Intervention Duration	Intervention Type	Outcome Type	Risk of Bias
Gerstel et al. 2013 [21]	129	Employees of a nursing agency	Metabolic syndrome	48 weeks	PA Intervention and Health Education Intervention (obesity, PA, diet)	Clinical Health Outcomes, Body Composition, PA, and Diet and Nutrition	M
Andersen et al. 2013 [22]	160	Programmers	Insufficient PA	9 weeks	PA Intervention and Health Education Intervention	Clinical Health Outcomes, Body Composition, and PA	M
Christensen et al. 2013 [23]	144	Employees of a nursing agency	Improve work efficiency	12 weeks	PA Intervention, Dietary Intervention (restricted diet), and Health Education Intervention (diet)	Attendance Rate, Absenteeism Rate, and Work-Related or Organizational Outcomes (Productivity)	M
Gussenhoven et al. 2013 [24]	1288	Programmer, hospital, and insurance company	Obesity	24 weeks	Health Education Intervention (PA and diet)	Work-Related (Absenteeism Rate, GLPD, and NLPD)	H
Weinhold et al. 2015 [25]	69	University lecturer	Diabetes	16 weeks	PA Intervention, Dietary Intervention, and Health Education Intervention	Clinical Health Outcomes, Body Composition, PA, and Diet and Nutrition	L
Mitchell et al.2015 [26]	300	Farm grower	Obesity	12 weeks	Health Education Intervention (PA)	Clinical Health Outcomes, Body Composition, PA, and Diet and Nutrition	M
Miller et al. 2015 [27]	70	University worksite among employees	Diabetes	16 weeks	PA Intervention and Health Education Intervention	Clinical Health Outcomes, Diet and Nutrition, PA, and Work-Related Indicators	M
Audrey et al. 2015 [28]	84	Office workers	Insufficient PA	8 weeks	PA Intervention and Health Education Intervention (PA)	PA and Work-Related Indicators	M
Solenhill et al. 2016 [29]	3876	Employees in the transport services	Improve lifestyle habits	36 weeks	Health Education Intervention (PA and diet)	Body Composition, PA, Diet and Nutrition, and Sleep Quality	M
Gregoski et al. 2016 [30]	54	Bank employees	Obesity	10 weeks	PA Intervention and Health Education Intervention (PA and diet)	Body Composition, PA, Diet and Nutrition, Sleep Habits, and Work-Related Indicators	H
Chandrasiri et al. 2016 [31]	81	Administrative office staff	Non-communicable diseases	12 weeks	Health Education Intervention (PA, diet, smoking cessation)	Clinical Health Outcomes and Body Composition	H
Hendriksen et al. 2016 [32]	502	Insurance company	Improve lifestyle habits	40 weeks	Health Education Intervention (PA mainly)	Clinical Health Outcomes, Body Composition, and Work-Related Indicators	H
Jamal et al. 2016 [33]	194	University lecturer	Obesity	12 weeks	Health Education Intervention (PA and diet)	Clinical Health Outcomes, Body Composition, PA, Diet and Nutrition, Depression and Anxiety, and Self-Efficacy	M
Balk-Moller et al. 2017 [34]	566	The Social Welfare and Health Care Sector	Obesity	38 weeks	Health Education Intervention (PA and diet)	Clinical Health Outcomes and Body Composition	H
Maylor et al. 2018 [35]	89	Office workers	Insufficient PA	8 weeks	PA Intervention and Health Education Intervention (PA, chronic disease)	Clinical Health Outcomes, Body Composition and PA	H
Viester et al. 2018 [36]	314	Construction workers	Overweight	24 weeks	Health Education Intervention (PA and diet)	Clinical Health Outcomes, Body Composition, PA, and Diet and Nutrition	M
Ing et al. 2018 [37]	217	The Social Welfare and Health Care Sector and academic institutions	Obesity	36 weeks	Health Education Intervention	Clinical Health Outcomes, Body Composition, PA and Physical Fitness, Diet and Nutrition, and Self-Efficacy	M
Kouwenhoven-Pasmooij et al. 2018 [38]	491	Police and hospital	Cardiovascular disease	24 weeks	Health Education Intervention (lifestyle education)	Clinical Health Outcomes, Body Composition, PA, Diet and Nutrition, and Work-Related Indicators	M
Oftedal et al. 2019 [39]	40	Shift workers	Improve lifestyle habits	4 weeks	Health Education Intervention (PA, diet, sleep)	Clinical Health Outcomes, Body Composition, PA, Diet and Nutrition, Sleep Quality, and Work-Related Indicators	M
Fang et al. 2019 [40]	98	High-tech industries	Obesity	12 weeks	PA Intervention	Clinical Health Outcomes, Body Composition, PA and Physical Fitness, Stress, and Work-Related Indicators	H
Edman et al. 2019 [41]	54	Healthcare system employees	Cardiovascular disease	12 weeks	Health Education Intervention (PA, diet, stress management)	Body Composition, PA, Stress, Depression, and Sleep Habits	H
Haufe et al. 2019 [42]	314	Automobile factory workers	Metabolic syndrome	24 weeks	PA Intervention	Clinical Health Outcomes, PA and Physical Fitness, Diet and Nutrition, Depression and Anxiety, and Work-Related Indicators	L
Stein et al. 2019 [43]	1000	Healthcare system employees and university worksite among employees	Obesity	96 weeks	Health Education Intervention	PA	M
Bonn et al. 2019 [44]	209	White-collar office employees	Improve lifestyle habits	12 weeks	PA Intervention and Health Education Intervention	Clinical Health Outcomes, Body Composition, PA, Diet and Nutrition, Sleep Quality, and Psychological Health Outcomes	M
Sareban et al. 2020 [45]	73	Hospital staff	Cardiovascular disease	48 weeks	PA Intervention	Clinical Health Outcomes, Body Composition, and PA	M
Linnan et al. 2020 [46]	553	Child care center	Improve lifestyle habits	24 weeks	Health Education Intervention	PA	M
Piao et al. 2020 [47]	121	Office workers	Insufficient PA	12 weeks	PA Intervention and Health Education Intervention (PA)	Sleep Quality and Habits	M
Reich et al. 2020 [48]	66	Doctor	Cardiovascular disease	48 weeks	PA Intervention	Clinical Health Outcomes, Body composition, PA and Physical Fitness	M
Haufe et al. 2021 [49]	314	Automobile factory workers	Metabolic syndrome	24 weeks	PA Intervention, Dietary Intervention, and Health Education Intervention (PA)	Clinical Health Outcomes, Body Composition, PA and Physical Fitness, Depression and Anxiety, and Work-Related Indicators	M
Neshteruk et al. 2021 [50]	250	Child care center	Insufficient PA	24 weeks	PA Intervention and Health Education Intervention (PA)	PA	M
Mamede et al. 2021 [51]	298	Administrative office staff	Insufficient PA	10 weeks	PA Intervention and Health Education Intervention	Clinical Health Outcomes, Body Composition, PA, and Work-Related Indicators	H
Noori et al. 2021 [52]	80	Fruit factory workers	Improve lifestyle habits	8 weeks	Health Education Intervention (PA, diet, mental health)	Clinical Health Outcomes, Body Composition, PA, Diet and Nutrition, Sleep Habits, Stress, and Work-Related Indicators	H
Stephenson et al. 2021 [53]	56	Office workers	Insufficient PA	8 weeks	PA Intervention and Health Education Intervention	Clinical Health Outcomes, Body Composition, PA, Diet and Nutrition, Psychological Health Outcomes, and Work-Related Indicators	L
Garcia Perez de Sevilla et al. 2021 [54]	24	University employees	Improve lifestyle habits	18 weeks	PA Intervention, Dietary Intervention, and Health Education Intervention (PA, diet)	Clinical Health Outcomes, PA, Diet and Nutrition, and Psychological Health Outcomes	M
Kong et al. 2022 [55]	955	Office workers	Obesity	48 weeks	PA Intervention, Dietary Intervention, and Health Education Intervention (PA, diet)	Body Composition, PA, Diet and Nutrition, and Self-Efficacy	M
Shiri et al. 2022 [56]	159	Healthcare system employees	Improve lifestyle habits	8 weeks	PA Intervention, Dietary Intervention, and Health Education Intervention (PA, diet)	Depression and Anxiety, Sleep Habits, and Work-Related Indicators	M
Bayerle et al. 2022 [57]	129	Automobile factory workers	Metabolic syndrome	24 weeks	PA Intervention and Health Education Intervention (PA)	Clinical Health Outcomes, Body Composition, PA, Depression, and Work-Related Indicators	M
Edwardson et al. 2022 [58]	756	Office workers	Insufficient PA	12 weeks	PA Intervention and Health Education Intervention (PA)	Clinical Health Outcomes, Body Composition, PA, Diet and Nutrition, Sleep Quality, Stress, Anxiety, and Work-Related Indicators	H
Silva et al. 2022 [59]	31	Office workers	Insufficient PA	16 weeks	PA Intervention	Clinical Health Outcomes, Body Composition, PA and Physical Fitness, Diet and Nutrition, and Stress	L
Brinkmann et al. 2023 [60]	206	Automobile factory workers	Diabetes	15 weeks	PA Intervention, Dietary Intervention, Health Education Intervention, and Smoking Cessation	Clinical Health Outcomes and Body Composition	H

PA interventions, which directly engaged employees in exercise or enhanced PA levels. “L” refers to low risk of bias; “M” refers to moderate risk of bias; “H” refers to high risk of bias.

## 3. Results

### 3.1. Study Selection

**Figure 1:**
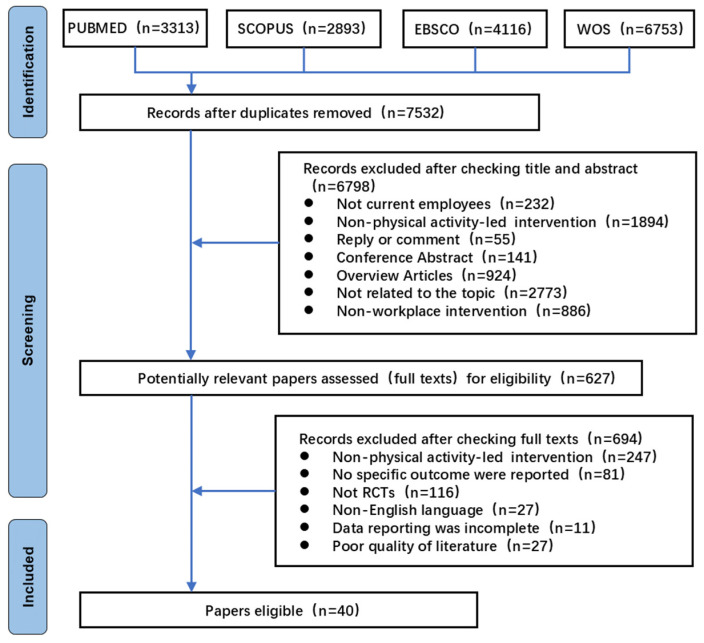
PRISMA flow diagram of the selection of studies.

This study ultimately included 40 RCTs investigating workplace interventions primarily focused on PA. The systematic literature selection process is illustrated in Figure 1. Of the 40 finally included studies, 22 were from PubMed, 10 from WOS, 6 from Scopus, and 2 from EBSCO/SPORTDiscus. This distribution shows the centralization trend of RCT-related PAWHI studies in biomedical and multidisciplinary databases.

### 3.2. Characteristics of the Samples

The analyzed studies encompassed a broad sample size distribution, totaling 14,414 participants across all included trials. The average intervention sample size was relatively large (mean = 360.35; median = 195.5). Participants were recruited from 11 countries: Sweden, the United States, Australia, China, Germany, Finland, the United Kingdom, Iran, Sri Lanka, Denmark, and the Netherlands. Intervention durations varied significantly, ranging from 4 weeks (shortest) to 96 weeks (longest). The most common intervention periods were 12 weeks (9/40 studies) and 24 weeks (24/40 studies). In terms of intervention design, 20 studies adopted multi-component intervention programs among single-component interventions: health education was the predominant approach (14/40 studies); physical activity interventions accounted for 5/40 studies.

### 3.3. Characteristics of the Interventions

#### 3.3.1. Educational Interventions

Health education represents the most prevalent strategy in workplace health interventions and serves as a critical supportive measure for PA interventions. In this review, 35 studies implemented health education as a primary supplementary approach in workplace health intervention programs.

The educational content encompassed diverse themes, primarily including the following: increasing PA and reducing sedentary behavior [36,58], improving dietary patterns and nutrition (including low-fat diets, Mediterranean dietary patterns, etc.) [21,24,27,32,34,41,43,46,54,55,56], weight management (including weight loss goal setting and energy intake and expenditure calculations) [21,23,27,33,34,37,43,45,55,58], knowledge regarding the benefits of PA for chronic disease management [31,35,38,40], mechanisms and methods of exercise for mental health promotion [21,27,28,32,38,41,50,51], and guidance on healthy lifestyle practices such as smoking cessation and alcohol management.

The health education interventions employed diverse delivery formats, including the following: educational manuals and video materials [24,27,37,39,50,54], online or in-person health assessments and personalized guidance by professionals (such as customized PA and dietary plans) [24,31,36,38,43], daily health behavior monitoring tools (including Polar heart rate monitors, pedometers, health diaries) [23,28,33,34,39,43,58], educational lectures or group meetings [21,27,31,32,35,37,45,50], multi-channel health information dissemination (including bulletin boards and posters) [35,46,50,55], internet and mobile technology-based health information delivery (including emails, text messages, WeChat groups) [29,34,43,50,55], and specialized health management applications or systems [28,39,47].

#### 3.3.2. Physical Activity Interventions

This systematic review analyzed 25 studies implementing workplace PA interventions, which directly engaged employees in exercise or enhanced PA levels. The findings indicate that these interventions primarily aimed to promote employee health through improved physical functioning. The interventions employed diverse approaches, including the following: (1) promoting active commuting, such as encouraging cycling or walking to and from work [21,28,35,42,45,48,50,51,55], (2) structured “work break exercises” during work breaks, such as workplace exercises [30], and (3) redesigning office environments by strategically placing waste bins and printers to increase routine PA [35,58]. Additionally, several studies implemented team-based collective activities, such as stair-climbing competitions [22,47] and step-counting challenges [30,35], while establishing incentive mechanisms and role models. As organizations increasingly prioritize employee health, various monitoring and exercise-facilitating devices were utilized to enhance motivation and accessibility, including pedometers [55], video monitoring systems [30], sit–stand workstations [58], and mobile applications or digital platforms [53,57] for exercise guidance and monitoring, effectively increasing PA levels.

#### 3.3.3. Dietary Interventions

This review examined 18 studies involving dietary and nutritional supportive interventions in workplace settings. Through systematic analysis, these interventions primarily comprised the following: (1) dietary and nutritional health education, such as food nutrient composition analysis and balanced diet guidance [21,23,24,25,27,49,60], (2) practical tool applications, including food measuring instruments [29], (3) metabolic monitoring, encompassing daily recording of energy intake and expenditure, and (4) dietary journals for the systematic documentation and analysis of individual eating behaviors [49], aimed at promoting rational, healthy eating and controlling low-fat dietary intake. (5) Additionally, dietary intervention studies employed various group participation strategies and workplace environment modification approaches. These included establishing nutrition and health-focused groups to foster peer motivation among members, organizing healthy eating competitions or challenges [55], implementing environmental interventions such as improving healthy food options in cafeterias and workplace supermarkets [55].

#### 3.3.4. Other Intervention Types

This review analyzed 4 studies involving smoking cessation [31,39,45,60] and alcohol reduction [39,44] interventions. Smoking cessation interventions primarily employed diverse health education approaches, including group promotional activities and individual face-to-face counseling, with some studies also utilizing nicotine replacement therapy, such as nicotine patches, to facilitate cessation. Regarding alcohol consumption issues, studies mainly implemented dietary education and self-monitoring strategies through food diaries to reduce excessive drinking behaviors. Additionally, 7 studies [32,41,43,45,52,56] explored stress reduction interventions for employees, predominantly employing mindfulness or meditation techniques, combined with breathing exercises and muscle relaxation training, enhanced by musical or verbal guidance. Notably, other non-medical intervention types emerged in the research, such as measures targeting improved sleep quality among employees [39,41].

### 3.4. Measures Used

#### 3.4.1. Clinical Health Outcomes

Clinical health outcomes refer to changes or results in patients’ health status following medical interventions or health management measures, typically used to evaluate treatment efficacy, disease progression, or the effectiveness of health intervention strategies. In this review, 27 studies assessed clinical health outcomes, primarily through patient-reported outcomes (PROs) and biological outcomes.

Patient-reported outcomes are data directly provided by patients regarding their health status, including symptoms, functional status, and quality of life. Twelve studies employed standardized self-administered questionnaires, primarily including the following: (1) the Symptom Checklist-90 (SCL-90) [21] for assessing mental disorders and psychological conditions; (2) quality of life-related scales, such as the 36-Item Short-Form Health Survey (SF-36) [38,42,45,48,54,57], the World Health Organization Quality of Life-BREF (WHOQOL-BREF) [27,33,40,59], and the EuroQol 5-Dimension 5-Level (EQ-5D-5L) [58], which comprehensively evaluate the health status and quality of life from different dimensions; and (3) the Symptom and Neurotoxicity Questionnaire (SNQ) [27], specifically designed for diagnosing neurological and musculoskeletal symptoms.

Biological outcomes are objective indicators obtained through laboratory tests or imaging examinations, which served as key assessment metrics in 18 studies. These biological outcomes primarily included the following: (1) cardiovascular-related indicators, such as blood pressure [22,25,27,32,34,35,36,37,40,45,57,58,60], electrocardiograms [48,57], and cardiovascular risk scores [45,48,60]; (2) metabolism-related indicators, including blood glucose [21,25,26,40,45,48,57] and blood lipids (e.g., total cholesterol, low-density lipoprotein, high-density lipoprotein, and triglycerides) [21,25,32,34,35,36,40,45,48,57,58,59,60]; and (3) inflammation and disease-specific markers, such as high-sensitivity C-reactive protein (hs-CRP) [60] as a predictor of inflammation and cardiovascular events, glycated hemoglobin (HbA1c) [58,60] for long-term glycemic control assessment, and liver fibrosis-related indices (FIB-4, APRI, and AST) [49] for evaluating the degree of hepatic fibrosis.

#### 3.4.2. Body Composition

Body Composition refers to the proportional distribution of various tissues, organs, and substances within the human body, serving as a critical indicator for assessing individual health status, nutritional condition, and disease risk. In this systematic review, 29 studies evaluated body composition-related outcomes. Among the reported body composition outcome parameters, the most frequently included metrics were as follows: (1) body mass index (BMI) [22,26,29,30,31,33,36,37,38,39,40,41,45,48,51,52,53,55,57,59,60], serving as a fundamental indicator for assessing overall obesity; (2) visceral fat area (VFA) or body fat percentage [21,32,34,40,42,45,48,55,58,59], used to evaluate fat distribution and accumulation patterns; and (3) waist-to-hip ratio [21,25,26,33,34,35,36,40,45,48,55,58,59,60], functioning as a predictor of central obesity and metabolic risk. Regarding body composition assessment methodologies, the most commonly employed techniques in these studies included Bioelectrical Impedance Analysis (BIA) [21,40,44,45,55,59], anthropometric methods (such as circumference measurements), and skinfold thickness measurements [48]. Notably, although dual-energy X-ray absorptiometry (DEXA) is considered the gold standard for body composition assessment, it was not utilized in any of the studies included in this review.

#### 3.4.3. Diet and Nutrition

This review analyzed dietary and nutritional outcome indicators from 19 studies, primarily designed to assess individual nutritional status, dietary behaviors, and their health impacts. The findings indicate that the core assessment framework for diet and nutrition encompasses dietary reference intakes and nutritional status classification, with specific indicators covering nutrient intake, nutritional status, dietary behaviors, and health outcomes, measuring changes in food variety, dietary intake volume, water consumption, health status, eating habits, and dietary perceptions.

Regarding assessment methodologies, studies predominantly employed standardized questionnaires and dietary recording techniques. Standardized questionnaire instruments included the following: (1) Food Frequency Questionnaire (FFQ) [21,25,27,39,44,45,59], assessing specific food intake frequency; (2) Weight Efficacy Lifestyle Questionnaire (WEL) [33], measuring individual self-efficacy in controlling diet across different situations; (3) Mediterranean Diet Adherence Screener (MEDAS) [53], evaluating adherence to the Mediterranean dietary pattern; (4) Australian Recommended Food Score (ARFS) [39], gauging dietary quality; (5) Health-Promoting Lifestyle Profile (HPLP) [52,54], assessing health behaviors and lifestyle; (6) Three-Factor Eating Questionnaire (TFEQ-R21) [44], measuring relationships between individual eating behaviors and emotional and cognitive factors; and (7) Alcohol Use Disorders Identification Test—Consumption (AUDIT-C) [29], screening for drinking behaviors and alcoholism risk.

Beyond questionnaire assessments, multiple studies implemented a 24 h dietary recall or dietary record methods (3-day or 7-day records) [26,30,36,37,38,55,58], documenting daily dietary intake in detail and integrating food energy tools or reference manuals to estimate caloric content, thereby promoting dietary self-monitoring and management [33,42].

#### 3.4.4. Physical Activity and Function

This systematic review identified 32 studies that evaluated outcome indicators related to PA levels and physical function changes, with assessment methods categorized into objective measurements and subjective assessments.

Objective measurement methods directly record PA data through specialized equipment or technologies, providing high-precision results suitable for individualized assessment and precise scientific research. These methods primarily include the following motion sensors: (1) accelerometers (e.g., ActiGraph and GENEActiv), typically worn on the waist, wrist, or ankle, capable of recording body acceleration and distinguishing low, moderate, and high-intensity PA [25,27,28,35,43,44,50,51,53,55,58,59]; (2) pedometers, used for recording daily step counts, simple to operate but unable to differentiate activity intensity [43,55]; (3) smart bands/watches, such as Fitbit, Garmin, and Apple Watch, capable of simultaneously monitoring the heart rate, step count, calorie expenditure, and activity duration [43,51]; and (4) heart rate monitors, which estimate energy expenditure and activity intensity through heart rate data, particularly suitable for assessing moderate-to-high-intensity activities [45,48].

Subjective assessment methods rely on individuals’ self-reporting or recall of PA patterns, applicable to large-scale epidemiological surveys and population studies. Standardized questionnaires employed in these studies include the following: Physical Activity Frequency Questionnaire (PAFQ) [21,38], International Physical Activity Questionnaire (IPAQ) [22,26,33,36,48], Global Physical Activity Questionnaire (GPAQ) [54], Freiburg Physical Activity Questionnaire (FPAQ) [49,57], Occupational Sitting and Physical Activity Questionnaire (OSPAQ) [58], Godin–Shephard Leisure-Time Physical Activity Questionnaire (GSLTPAQ) [55], and the Active Australia Questionnaire (AAQ) [39], used to evaluate activity frequency, intensity, and duration. Additionally, some studies utilized comprehensive scales such as the Health-Promoting Lifestyle Profile-II (HPLP-II) [52], assessing PA patterns during specific time periods through its PA dimension. Multiple studies also implemented PA logs to document participants’ exercise habits and training elements in detail, ensuring the rational implementation of exercise interventions [29,30,43,45,46,51].

Beyond directly increasing employee PA levels, studies also measured intervention effectiveness by assessing employees’ physical fitness and functional capacity, including specialized tests for muscle strength, cardiorespiratory endurance, and flexibility [37,40,41,42,59].

#### 3.4.5. Sleep Habits and Quality

In this review, eight studies evaluated sleep status as an outcome indicator. These studies primarily employed subjective assessment methods, including standardized scales such as the Pittsburgh Sleep Quality Index (PSQI) [39,58], the Karolinska Sleep Scale (KSS) [29], and Numerical Rating Scales (NRSs) [41]. Additionally, several studies utilized sleep status self-recording forms or online sleep monitoring software systems to conduct multidimensional assessments of employees’ sleep habits and sleep quality [30,44,56]. These assessment tools collectively formed a comprehensive indicator system for evaluating the impact of workplace interventions on employee sleep health.

#### 3.4.6. Psychological Health Outcomes

Based on a comprehensive integration of research analyses, workplace health interventions predominantly focused on PA demonstrate a concentrated emphasis on three key dimensions of psychological health outcomes: psychological stress management, depression and anxiety symptomatology, and exercise-related psychological factors (such as self-efficacy and motivational adherence). These interventions operate through diverse mechanisms to positively influence the psychological well-being of working populations.

(1) Psychological stress assessment: Five studies focused on the evaluation of psychological stress and stress management. The most commonly utilized instrument for assessing individual psychological stress was the Perceived Stress Scale (PSS) [41,45,58,59], while work environment-related stress was evaluated using the Occupational Stress Scale [40]. Additionally, the Health-Promoting Lifestyle Profile-II (HPLP-II) was employed to assess participants’ stress management levels [52].

(2) Emotional state assessment: Seven studies addressed the evaluation of depression, anxiety, and burnout. These studies primarily employed the Hospital Anxiety and Depression Scale (HADS) [42,49,57,58], the Generalized Anxiety Disorder-7 (GAD-7), and the Patient Health Questionnaire-9 (PHQ-9) [56] for relevant assessments. Furthermore, the Automatic Thoughts Questionnaire (ATQ) was used to evaluate negative cognitive patterns [33], while Numerical Rating Scales (NRSs) for fatigue and pain were utilized to assess individual levels of fatigue and pain during specific timeframes [41].

(3) Exercise-Related Psychological Factor Assessment: Three studies examined exercise self-efficacy and exercise motivation, primarily through employee self-report questionnaires. Specific assessment tools included the Weight Efficacy Lifestyle Questionnaire (WEL) [33] and the Self-Efficacy for Exercise Scale (SEE) [37,55], which were used to evaluate participants’ exercise motivation and self-efficacy levels.

#### 3.4.7. Work-Related or Organizational Outcomes

This review included 16 studies that evaluated work-related outcome indicators using multiple standardized assessment tools. These instruments can be categorized as follows: (1) work engagement assessment, such as the Utrecht Work Engagement Scale (UWES) [58], measuring employees’ energy, dedication, and absorption in their work; (2) work limitation assessment, including the Work Limitations Questionnaire (WLQ-8) [38,58] and the Work Productivity and Activity Impairment Questionnaire (WPAI-GH 2.0) [28], evaluating barriers and limiting factors encountered during work processes; (3) work ability assessment, primarily conducted through the World Health Organization Health and Work Performance Questionnaire (WHO-HPQ) [58] and the Work Ability Index (WAI) [38,42,49,56,57]; (4) employee vitality and performance assessment, such as the Energy and Performance Scan (EPS) [32], comprehensively evaluating employees’ vitality, work performance, and attendance; and (5) job satisfaction assessment, employing the Minnesota Satisfaction Questionnaire (MSQ) [40] to measure employees’ satisfaction with their work. Additionally, multiple studies assessed work efficiency through objective indicators, including weekly workday counts and working hours statistics [51,52], Lost Productivity Days (LPDs) [24], as well as records of sick leave days, attendance rates, and absenteeism rates [23,27,30,32,53].

### 3.5. Overall Effects of the Interventions

#### 3.5.1. Clinical Health Outcomes

Compared to baseline levels, intervention results from studies included in this review demonstrated significant improvements in employees’ overall health status and quality of life, as assessed through standardized self-report questionnaires such as the WHOQOL-BREF [33,40,59] and SF-36 [21,38,42,45,48,54]. Further analysis indicated that PAWHIs also yielded positive changes in clinical indicators, including significant reductions in metabolic syndrome scores [42] and cardiovascular risk scores [60], as well as improvements in FIB-4, APRI, and AST [49]. Total cholesterol, low-density lipoprotein, high-density lipoprotein, triglycerides, and blood pressure monitoring, which served as the most common health assessment measures, showed positive changes across multiple studies [21,22,25,37,40,45,48,49,59,60]. Notably, among the 27 studies involving clinical health outcomes, 15 studies reported statistically significant health improvements [21,22,25,33,37,38,40,42,45,48,49,54,58,59,60], indicating that workplace lifestyle interventions have a significant positive impact on employees’ clinical health indicators.

#### 3.5.2. Body Composition

The systematic review identified 16 studies demonstrating significant improvements in at least one body composition parameter following workplace health management interventions. These improvements encompassed various anthropometric measures, including body weight, BMI, waist circumference, hip circumference, waist-to-height ratio, body fat percentage, and visceral adipose tissue [21,22,25,26,30,33,34,36,37,38,40,41,49,55,59,60].

#### 3.5.3. Diet and Nutrition

Among the 19 studies reporting nutrition or healthy eating outcomes, 12 studies demonstrated significant improvements in dietary patterns or nutritional status following interventions [21,25,26,27,29,33,36,39,42,52,54,55], primarily manifested as transformations in dietary concepts, the optimization of the dietary structure, and the enhancement of dietary scores. Workplace dietary interventions facilitated the development of scientifically sound eating habits among employees through comprehensive strategies, including nutrition education, healthy menu design, and environmental optimization. Specific improvement indicators encompassed increased dietary quality scores [42,52,54], reduced daily caloric intake [21,25,55], optimized dietary structure [25,26,27,29,33,42], and improved hydration habits coupled with decreased sugar-sweetened beverage consumption [36]. Notably, in five of these studies, researchers integrated PA interventions with dietary interventions, incorporating individualized exercise goals [25] and increasing the frequency of aerobic activities such as walking or cycling [21,27,42,55], thereby significantly enhancing the effectiveness of comprehensive health interventions. However, some studies also indicated that the efficacy of dietary and nutritional interventions was constrained by multiple factors during implementation, including insufficient employee compliance, varying levels of cafeteria management quality, and cost control pressures (for instance, some employees opted for takeout or fast food due to busy work schedules), which to some extent diminished the effectiveness of the interventions.

#### 3.5.4. Physical Activity and Function

Twenty-six studies demonstrated significant improvements in PA-related outcomes following interventions [21,22,25,27,29,33,35,36,37,38,39,40,41,43,45,46,48,49,50,51,52,53,54,55,57,58]. Among these studies, improvements in PA were manifested across multiple dimensions: increased activity frequency [37,41], extended duration (reflected by increased step counts, extended cycling distances, and increased daily intervention time) [21,27,45,51,55], and enhanced exercise intensity (transitioning from light-to-moderate or vigorous activity) [21,51]. Additionally, the interventions significantly improved employees’ cognition regarding PA [33,52,54], enhanced motivation for exercise participation [29,37,55], and effectively reduced sedentary behavior [35,54,58]. The majority of studies employed PA assessment questionnaires or objective tools such as accelerometers to evaluate intervention effects, confirming significant improvements in PA levels [22,25,27,29,33,35,36,38,43,46,49,50,53,54,55,57,58]. Notably, following workplace healthy lifestyle interventions, employees’ physical fitness parameters also exhibited marked improvements, including increased vital capacity, enhanced aerobic endurance, improved strength, and better flexibility [37,40,41,48,49,57].

#### 3.5.5. Sleep Habits and Quality

Among all studies included in the analysis, only two studies reported positive effects of PAWHI on employees’ sleep quality [46,56]. This notably low proportion may reveal the multifactorial etiology of sleep disorders. Employees’ sleep quality is subject to synergistic regulation by multidimensional factors, including occupation-related factors (such as workload, occupational stress, and work environment), psychosocial factors (such as family relationships and life events), and individual health status (such as anxiety, depression, and other psychological issues). PA-only interventions may not be sufficient to address the multifactorial causes of sleep disturbances. Therefore, their effects on sleep quality may be limited. This finding suggests that when designing workplace health promotion programs, it may be necessary to integrate diversified strategies, including psychological interventions, environmental optimization, and lifestyle adjustments, to more effectively address employee sleep problems.

#### 3.5.6. Psychological Health Outcomes

Among the included studies, 14 recorded and evaluated the impact of interventions on psychological status. Of these, five studies focused on psychological stress and coping abilities, with four demonstrating significant improvements in stress-related indicators following intervention, primarily manifested as enhanced stress source identification, reduced stress levels, improved stress management skills, and optimized coping strategies [40,41,52,58]. Additionally, seven studies addressed negative emotional states such as depression and anxiety, with three confirming that workplace health interventions effectively alleviated employees’ depressive or anxiety symptoms [42,56,57]. Furthermore, three studies investigated the impact of interventions on self-efficacy and exercise motivation, with one observing significantly improved exercise self-efficacy among employees post-intervention [33]. These results suggest that workplace health interventions have potential for improving employees’ psychological health, though effects vary across different psychological domains, indicating that future intervention designs need to be customized according to specific psychological health dimensions.

#### 3.5.7. Work-Related or Organizational Outcomes

In this review, 16 studies included assessments of at least one work-related or organizational outcome. Among these, seven studies demonstrated statistically significant positive impacts of PAWHIs on work-related outcomes, primarily reflected in enhanced productivity and work ability [23,32,42,56], increased attendance rates [27,30,32], and improved job satisfaction [40]. However, several studies also revealed the limitations of such interventions, with some reporting that interventions failed to deliver expected work-related benefits to organizations while incurring relatively high implementation costs [24], and isolated studies even observed slight decreases in productivity following intervention [38]. Notably, none of the studies included in this analysis reported intervention effects on long-term organizational indicators, such as early retirement intentions, employee retention rates, or staff turnover rates, suggesting that future research needs to expand the range of outcome measures to comprehensively evaluate the organizational benefits of workplace health promotion programs.

### 3.6. Success Rate of the Interventions

The significantly improved outcome measures from the included studies were systematically compiled, with the specific improvement status for each outcome type detailed in Table 4.

This study systematically evaluated the impact of PAWHIs on multidimensional health outcomes, with results summarized in Table 5. Analysis revealed significant variations in intervention effectiveness across different health domains. Health domains with relatively high intervention success rates included PA and physical function improvement (81.3%), stress management (80.0%), and dietary and nutritional status (63.2%). Domains with moderate intervention effectiveness encompassed clinical health indicators (55.6%), body composition (55.2%), work-related outcomes (43.8%), and depressive and anxiety symptoms (42.9%). Domains showing comparatively lower intervention effects included self-efficacy enhancement (33.3%) and sleep quality improvement (28.6%). Notably, the number of intervention studies addressing psychological health dimensions such as stress management, depressive and anxiety symptoms, and self-efficacy was relatively limited, potentially affecting the robustness of related conclusions. This suggests that future research should further expand sample sizes in these domains to obtain more reliable evidence.

### 3.7. Risk-of-Bias Results

The risk of bias for all included studies was independently assessed by two reviewers using the Cochrane risk-of-bias assessment tool, with discrepancies resolved through reassessment by a third reviewer and consensus reached through discussion.

**Figure 2:**
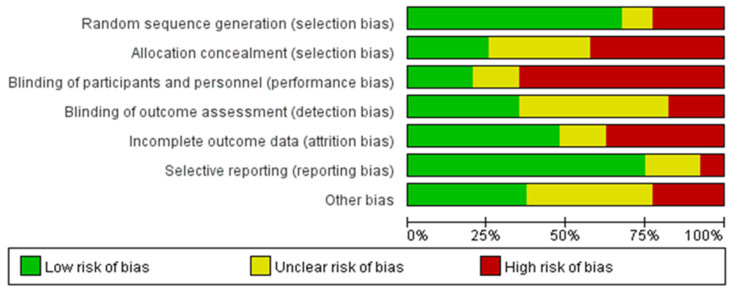
Estimated risk of bias across all studies (1).

**Figure 3:**
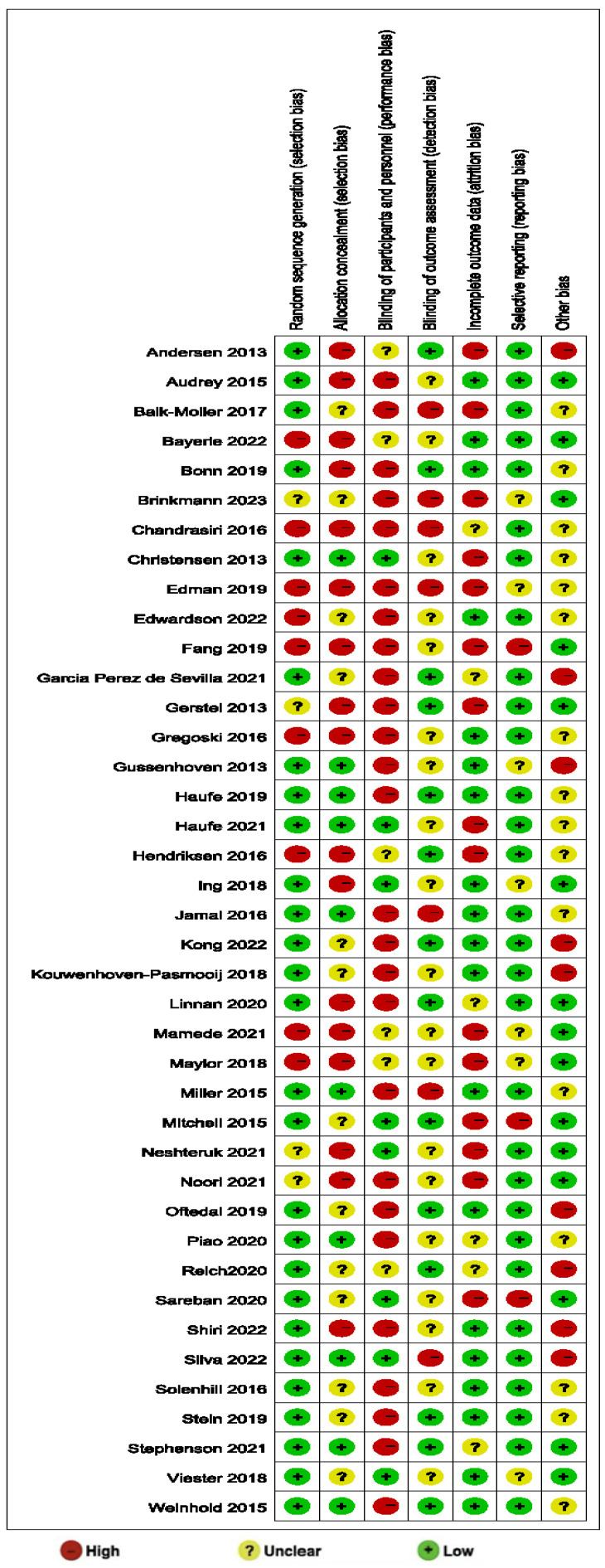
Estimated risk of bias across all studies (2) [21,22,23,24,25,26,27,28,29,30,31,32,33,34,35,36,37,38,39,40,41,42,43,44,45,46,47,48,49,50,51,52,53,54,55,56,57,58,59,60].

Comprehensive bias risk analysis revealed that among all included RCTs (n = 40), the inadequate blinding of participants and/or personnel represented the most prevalent source of high-risk bias (26/40, 65.0%). Other high-risk biases, in descending order of frequency, included insufficient or absent allocation concealment (17/40, 42.5%), incomplete outcome data (15/40, 37.5%), inadequate random sequence generation (9/40, 22.5%), other sources of bias (9/40, 22.5%), and a lack of outcome assessment blinding (7/40, 17.5%), while selective reporting demonstrated the lowest proportion of high-risk bias (4/40, 10.0%). Notably, a substantial number of studies (19/40, 47.5%) inadequately described the blinding status of outcome assessment, resulting in an “unclear” risk rating for this dimension. Beyond issues with participant and personnel blinding, an insufficient description of allocation concealment and randomization methods constituted common methodological limitations. Regarding outcome assessment blinding, as most studies employed objective measurement indicators (such as blood biochemical parameters, electrocardiographic examinations, and standardized exercise tests), the practical impact of detection bias may be relatively limited. The final assessment indicated that only seventeen studies (42.5%) achieved four or more “low-risk” ratings across the seven dimensions, and only four studies (10.0%) achieved five or more ‘low-risk’ ratings, suggesting that the overall methodological quality of included studies requires improvement. The risk-of-bias rating results of each study are shown in Table 3. The specific results are shown in Figure 2 and Figure 3.

## 4. Discussion

### 4.1. Summary of the Characteristics of the Studies

The literature included in this study shared the following key characteristics: the study population comprised employed adults aged 18 to 65 years, with particular emphasis on those in unhealthy working conditions; all studies employed RCT methodology; interventions featured PAWHI; and intervention settings were restricted to occupational environments. The intervention measures primarily included the following: (1) diversified health education (encompassing PA guidance, dietary and nutritional knowledge, exercise-based weight reduction principles, mechanisms of exercise for chronic disease improvement, strategies for exercise-promoted mental health, and smoking/alcohol cessation guidance); (2) structured exercise organization and PA promotion; (3) dietary pattern adjustment and nutritional intervention; (4) meditation and mindfulness training; and (5) tobacco and alcohol dependency behavioral interventions. The main outcome measures evaluated in these studies addressed seven health-related domains: (1) clinical health indicators (e.g., blood pressure, blood lipids, blood glucose); (2) body composition parameters (e.g., weight, BMI, body fat percentage); (3) dietary behavior and nutritional status; (4) sleep quality and patterns; (5) psychological stress levels; (6) depressive and anxiety symptoms; and (7) work-related performance metrics.

### 4.2. Successfulness of Interventions

This study systematically evaluated PAWHI, revealing significant health benefits, relatively low implementation costs, and sustainable long-term health promotion effects. In addition, due to high heterogeneity in intervention design, duration, and outcome measurement, meta-analysis was not feasible.

#### 4.2.1. Clinical Health Outcomes

Among 27 RCTs assessing clinical health outcomes, 15 (55.6%) reported significant improvements. Recent research confirms that integrated interventions combining dietary adjustments, increased PA, and psychological support effectively improve metabolic health indicators such as blood glucose and lipid profiles, thereby reducing the risk of cardiovascular disease (CVD) and diabetes [39,61]. Systematic reviews further support workplaces as ideal controlled environments for increasing moderate-to-vigorous physical activity (MVPA) levels and enhancing cardiometabolic health [62].

However, some studies indicate that because these interventions are typically implemented uniformly at the workplace level rather than tailored to individual differences, their impact on improving employee health status and reducing healthcare costs may be limited. An 18-month intervention study involving 32,974 employees showed limited improvements in self-reported health status, cholesterol, and blood pressure [63]. This suggests that despite positive progress in improving clinical health indicators through workplace health interventions, attention must be paid to the long-term effectiveness and sustainability of intervention measures. Clinical practice indicates that single health outcome indicators have limited predictive ability, necessitating the integration of multiple indicators to optimize risk assessment [64]. Additionally, successful implementation requires the thorough consideration of employee engagement and personalized needs [65].

#### 4.2.2. Body Composition

Among the 29 studies that looked at body composition-related outcome indicators, 16 of them, which accounted for 55.2%, showed significant improvements. Intervention duration emerged as a critical factor influencing effectiveness, with short-term interventions (<3 months) potentially yielding only temporary improvements [66]. Research indicates that the most effective workplace exercise interventions combine at least 4 months of supervised guidance, moderate-intensity aerobic exercise, and strength training [67].

The combination of aerobic exercise and strength training demonstrates synergistic effects in improving body composition: aerobic exercise (such as moderate-intensity continuous training) effectively reduces total fat mass (TFM) and visceral adipose tissue (VAT) [68], while strength training helps maintain or increase fat-free mass (FFM), thereby optimizing the muscle-to-fat ratio. Although high-intensity interval training (HIIT) offers advantages in time efficiency, its fat reduction effects show no significant difference compared to moderate-intensity aerobic exercise [69].

Notably, the diversity of body composition assessment tools (such as DXA, BIA, and skinfold thickness) may introduce measurement bias. DXA precisely differentiates fat and muscle distribution, while BIA is significantly affected by fluid changes and may overestimate fat-free mass [70]. Similarly, waist circumference as a proxy indicator for visceral fat, though operationally simple, correlates with VAT in ways that are influenced by gender and ethnicity, suggesting the need to combine multimodal measurements to enhance result reliability [71].

#### 4.2.3. Diet and Nutrition

Workplace dietary interventions are influenced by multiple factors, including work schedules, environment, and culture [72]. This study shows a 63.2% success rate for dietary interventions. As an auxiliary intervention measure in workplaces, dietary interventions significantly improve employees’ dietary quality and body mass index [73]. Workplace healthy eating policies and management measures, such as cafeteria renovations, healthy food options, nutritional labeling, and environmental cues, effectively support employees in forming healthy lifestyles [74].

Single dietary interventions often have a limited impact, whereas combining them with exercise and behavioral strategies tends to yield stronger results. A workplace dietary intervention study involving 602 employees found that two years of intervention influenced employees’ food choices to some extent, but the single dietary intervention model did not significantly improve employee weight status [75]. In contrast, RCTs confirmed that intensive lifestyle interventions combining diet and exercise significantly improved blood glucose control, weight management, and cardiopulmonary function in type 2 diabetes patients when implemented in the workplace [76]. Gepner et al. integrated dietary interventions with workplace exercise interventions, achieving clinically meaningful improvements in body composition [77]. Therefore, workplace dietary interventions should use combined strategies, particularly those integrating physical activity.

#### 4.2.4. Physical Activity

Comprehensive assessment indicates that increased PA levels and healthy exercise habits demonstrate significant intervention effects in improving employee body composition, functional status, disease management, and work-related indicators. The central role of PA interventions in workplace health promotion cannot be overlooked [78,79]. Numerous studies confirm that PA interventions not only bring significant health benefits to employees but also improve work performance and reduce medical costs [21,25,80].

PA interventions provide both immediate short-term benefits, such as significantly increased PA levels [81] and preliminary improvements in health awareness and behavior [82,83], as well as long-term health benefits through sustained exercise habit formation, including overall physical health improvements [84,85], body composition optimization [86], enhanced mental health and cognitive function [87], and increased job satisfaction and engagement [88]. PA interventions address not just single health issues but serve as comprehensive strategies for addressing multidimensional health challenges [89].

The team incentives and social attributes of workplace PA interventions show significant advantages in promoting employee participation [90,91,92]. Research indicates that team-based economic incentives (such as team rankings and financial rewards) significantly increase employees’ daily step counts, while “supervisory” information in team communications (such as encouraging more active member participation) correlates significantly with individual step count increases [93]. Additionally, mobile health technologies in workplace interventions provide convenient means for self-monitoring and PA level assessment [94].

Based on the literature review, successful PAWHIs should include the following core elements: (1) comprehensive interventions at the workplace level (including text messages, online platforms, etc.), providing personalized health guidance for employees; (2) establishing health lifestyle management systems such as platforms for data collection and management; (3) conducting health education and promotion through multiple channels such as training meetings, website resources, and educational materials; (4) focusing on behavioral habit improvement as the core objective, optimizing work routines (such as increasing standing, stretching, and scheduled hydration); (5) promoting self-management, reducing management costs, enhancing employee autonomy, and stimulating intrinsic motivation and self-efficacy (such as setting personalized PA goals); (6) organizing sports events and challenges with incentive mechanisms; and (7) implementing comprehensive monitoring and feedback systems to ensure intervention quality.

#### 4.2.5. Mental Health and Self-Efficacy

Research indicates that PAWHIs (including regular exercise, balanced diet, and sleep management) help reduce stress and improve emotional stability [95]. A systematic review assessed the effectiveness of organizational-level workplace mental health interventions on stress, burnout, non-clinical depression and anxiety symptoms, and well-being among construction workers [96]. Most studies show that workplace interventions demonstrate mild-to-moderate effects in improving mental health conditions (such as burnout, insomnia, and stress) and enhancing positive mental health (such as well-being) [97,98].

Specific studies emphasize that short-duration activities such as lunchtime walks or yoga help employees temporarily escape high-pressure work environments and restore psychological resilience [99,100,101]. However, these benefits depend on sustained participation, and their effects are significantly influenced by individual compliance. One study applying Bandura’s self-efficacy model to explore employee participation in lifestyle intervention occupational rehabilitation programs found that enhanced self-efficacy helps participants more effectively cope with work stress, improve health behaviors, and enhance work performance [102]. Another study indicated that workplace health education and mental health promotion significantly increase employee self-efficacy, effectively promoting weight management and health behavior change [103].

It should be noted that this study included a limited number of studies on self-efficacy and depression/anxiety-related outcomes. Constrained by the overall research scale, the strength of the related evidence is relatively insufficient, suggesting future research should further expand sample sizes to obtain more reliable evidence. Future studies should use longitudinal designs with control groups to clarify causal relationships and mechanisms of change, particularly for self-efficacy and psychological outcomes.

#### 4.2.6. Work-Related Outcomes

Work-related outcomes in this study showed moderate improvement. Among eighteen studies evaluating work-related outcomes, eight demonstrated improvement in one or more indicators: four studies observed enhanced productivity and work ability, three found increased attendance rates, and one showed increased employee job satisfaction.

Currently, no clear consensus exists regarding the effectiveness of workplace interventions in reducing absenteeism. A systematic review of positive workplace interventions for reducing sick leave concluded that existing evidence is insufficient to support the effectiveness of such interventions for reducing sick leave [104]. However, another systematic review on the effectiveness of PAWHIs for work-related and health-related outcomes (musculoskeletal diseases, mental health issues, or other health conditions) found that for the musculoskeletal disease subgroup, such interventions effectively reduce sick leave [105]. Similarly, a systematic review indicated that comprehensive low-back pain interventions positively impacted absenteeism, cost control, and the prevention of new-onset low-back pain [106].

Research shows that work ability typically declines by 0.5–0.7 percentage points annually with age, and improvements in work ability can be viewed as an important indicator of corporate economic benefits [107]. A meta-analysis confirmed that workplace interventions improve employee work ability and increase productivity, although the magnitude of effects is relatively small [108].

#### 4.2.7. Sleep Quality

The sleep improvement results in the included studies demonstrated relatively limited efficacy, with only two out of seven studies (28.6%) reporting statistically significant improvements. This finding contrasts with general research on physical activity (PA) and sleep, where appropriate PA typically shows positive effects on sleep quality—particularly in enhancing deep sleep, reducing sleep latency, and improving sleep efficiency [109,110]. However, individual variability and the complex regulatory mechanisms of sleep, combined with external influencing factors, may explain the inconsistent intervention effects. In the two studies that reported significant improvements, participants were office workers with fixed schedules and predominantly sedentary behaviors. In the two studies with significant improvements, the inclusion of educational and structured PA components may have contributed to positive outcomes, though broader conclusions are limited by the small sample size. Existing research on sleep and PA suggests that achieving a certain exercise intensity is crucial: moderate-intensity PA has been shown to significantly enhance sleep quality, whereas low-intensity PA yields minimal effects [111,112]. Additionally, age is a key moderating factor, with more pronounced sleep benefits observed in older adults and children [113]. Additionally, balanced nutrition positively affects sleep quality [109,114,115]. Research confirms that diets containing the appropriate amounts of protein, carbohydrates, and healthy fats are crucial for maintaining sleep quality [116]. To improve sleep quality, it is recommended to prioritize foods containing complex carbohydrates, a low glycemic index, low glycemic load, and high fiber content, while avoiding processed foods rich in saturated fatty acids [117].

Due to the limited number of included studies, further investigation is needed to clarify the effectiveness of physical activity-led workplace health interventions (PAWHIs) on employee sleep outcomes. Future research should explore differential effects by age, occupational demands, and baseline activity levels to clarify which subgroups benefit most from PAWHI in sleep-related outcomes.

### 4.3. Risk-of-Bias Results

The risk-of-bias assessment revealed methodological limitations in the included studies. A high prevalence of performance bias (65.0%, 26/40 studies) due to the inadequate blinding of participants and personnel was observed. The insufficiency of blinding is largely limited by the characteristics of the intervention measures. If the intervention involves visible health management activities (such as fitness classes and dietary guidance), it will be difficult to set blinding methods for both the intervener and the participants.

Allocation concealment and random sequence generation were insufficiently described in 42.5% and 22.5% of studies, respectively, compromising baseline comparability. Among the included PAWHI studies, some did not strictly implement the randomization method. They merely grouped companies or departments as a whole instead of randomly assigning individuals.

It is worth noting that in selective reporting, the proportion of high-risk situations is the lowest, which to some extent reduces the misleading conclusions caused by “selective emphasis”. Meanwhile, most of the primary outcome measures included in the studies were objective measures. Compared with subjective measures (such as self-reported stress or dietary habits), objective outcome evaluations (such as blood biomarkers and body composition) had the potential to offset the risk of bias because these measurements were less likely to produce explanatory variability.

Only 10.0% of studies met the ≥5 low-risk criteria, underscoring the need for improved methodological rigor in future workplace intervention trials. Emphasis should be placed on the transparent reporting of randomization, allocation concealment, and blinding protocols, particularly for studies relying on subjective outcomes. The integration of objective biomarkers and standardized tools could further enhance reliability.

This aligns with the review’s broader finding that PAWHI efficacy is robust for objective outcomes but more variable for subjective domains, where bias risks are higher. Especially in small-sample studies, attention should be paid. Future research should address these limitations to strengthen evidence quality.

### 4.4. Study Limitations and Prospects

Several notable limitations exist in the current study: First, PAWHIs are typically implemented uniformly at the organizational level. While this approach facilitates workplace culture transformation and provides extensive social support networks for health behavior change, the implementation across diverse demographic profiles, geographical regions, and employment contexts makes it challenging to precisely identify the potential determinants of outcome heterogeneity.

Second, the systematic reporting of organizational support levels and employee engagement metrics is conspicuously insufficient. Existing evidence suggests that active employee participation and strong management support are key predictors of intervention success; therefore, exploring strategies to optimize employee engagement and secure sustained management commitment to strengthen intervention effectiveness should be prioritized in future research.

Furthermore, current research indicates that there is limited attention paid to the cost-benefit analysis of the intervention. Although most research confirms the positive impacts of interventions on health indicators, their economic cost-effectiveness remains incomprehensively evaluated, particularly regarding long-term return on investment. Future research should incorporate economic evaluation methodologies to provide decision-makers with more comprehensive investment value assessment criteria.

Tension exists between intervention universality and personalized customization. Different employee subgroups (based on age cohorts, health status, job nature, and occupational strata) likely require differentiated intervention strategies. Consequently, determining how to maintain core intervention elements while making appropriate adjustments according to target population characteristics to enhance intervention precision and effectiveness represents a critical area requiring in-depth exploration. Concurrently, to achieve broader and more sustainable health intervention outcomes, future program designs should incorporate greater personalization, enhance long-term sustainability, and secure more comprehensive organizational support.

Finally, in the analysis of the intervention effect, future studies should prioritize standardized, objective measurement tools (e.g., DEXA for body composition and accelerometers for PA) to minimize methodological variability and enhance cross-study comparability.

## 5. Conclusions

This systematic review provides a comprehensive evaluation of PAWHIs in terms of specific intervention strategies and their multidimensional effects. The findings demonstrate that such workplace interventions exhibit varying degrees of effectiveness in improving employee health status, optimizing body composition, enhancing physical function, promoting mental well-being, and improving work-related outcomes The results of the intervention success rate indicate that, particularly for physical activity (81.3%), stress management (80.0%), and diet/nutrition status (63.2%), this confirms PAWHI have significant efficacy in these specific assessment indicators.

In conclusion, PAWHI represents an effective intervention strategy for enhancing employee health, fostering healthy lifestyle habits, and improving work performance. It offers corporate leaders innovative approaches to workforce management and employee health improvement.

## Figures and Tables

**Table 1:** The terms used in the search process.

MeSH	Entry Terms
Term #1: Physical Activity	Physical Activities; Exercise; Exercises; Physical Exercise; Physical Exercises; Acute Exercise; Acute Exercises; Aerobic Exercise; Aerobic Exercises; Isometric Exercise; Isometric Exercises; Exercise Training; Exercise Trainings
Term #2: Occupational Group	Occupational Groups; Employee; Employees; Personnel; Worker; Workers
Term #3: Workplace	Job Site; Job Sites; Work Location; Work Locations; Worksite; Worksites; Work Place; Work Places; Work-Site; Work Site; Work-Sites

**Table 2:** Inclusion and exclusion criteria based on the PICOS framework.

PICOS	Inclusion	Exclusion
Population	(1) Currently employed individuals; (2) Employees with suboptimal work habits, such as prolonged sitting, insufficient PA, high work-related stress, or physically demanding labor; (3) Adults aged 18–65 years.	(1) Non-employed individuals; (2) Special occupational groups (e.g., professional athletes and military personnel), with findings not generalizable to general workplace populations.
Intervention	(1) Workplace interventions primarily centered on PA; (2) Intervention themes may address the following: improvement of health risk factors, including overweight/obesity, dietary issues, insufficient PA, sleep problems, or tobacco/alcohol use; alleviation of disease symptoms, such as type 2 diabetes, hypertension, stroke, chronic heart disease, cancer, etc.; reduction in psychological distress, depression, anxiety, and other negative emotional states, with an enhancement in overall well-being; improvement in productivity, organizational performance, and reduction in absenteeism.	(1) Non-PA-led interventions; (2) Poorly described PA interventions (e.g., vague references to “exercise” without details on frequency, intensity, or type); (3) Intervention duration <4 weeks; (4) Interventions implemented in non-workplace settings (e.g., community and home).
Comparison	(1) Control groups receiving either no intervention or non-PA interventions; (2) No significant differences between intervention and control groups at baseline.	(1) Lack of a control group or mismatched control designs; (2) Significant baseline differences between intervention and control groups.
Outcom	Studies must include at least one of the following outcome categories: (1) Clinical health outcomes: blood pressure, heart rate, electrocardiogram measurements, blood biomarkers (e.g., glucose, lipids), incidence rates, and pain or disease status assessments; (2) Body composition outcomes: weight, BMI, waist circumference, other anthropometric measurements; (3) PA levels; (4) Physical function: muscular strength, aerobic endurance, joint mobility, etc.; (5) Sleep quality and habits; (6) Dietary behavior outcomes; (7) Psychological health: stress, anxiety, depression, burnout, self-efficacy measures; (8) Work-related outcomes.	(1) Absence of baseline or endpoint data; (2) Failed to report core outcomes related to PA or health (for example, only focusing on economic indicators).
Study Design	Randomized controlled trials.	Non-randomized designs, including observational studies, case reports, or narrative/systematic reviews.

**Table 4:** Success rates of interventions based on statistical significance.

Author/Year	Significantly Improved Outcome Measures After the Intervention (*p* < 0.05)
Gerstel et al. 2013 [21]	Clinical health outcomes: SBP, LDL-C, total C/HDL-C, HDL-C, fasting glucose, and mental component score of SF-36; body composition: body weight, BMI, and fat mass; diet and nutrition: total caloric intake (kcal/d); physical activity and function: low-intensity physical activity
Andersen et al. 2013 [22]	Clinical health outcomes: SBP and DBP; body composition: weight, BMI, and body fat percentage; physical activity and function: aerobic fitness (mL/min/kg)
Christensen et al. 2013 [23]	Clinical health outcomes: WHOQOL-BREF; work-related or organizational outcomes: productivity
Gussenhoven et al. 2013 [24]	Level of physical activity
Weinhold et al. 2015 [25]	Clinical health outcomes: FBG; body composition: body weight; diet and nutrition: the intake of dietary fiber and fats; level of physical activity
Mitchell et al. 2015 [26]	Body composition: weight, BMI, and waist; diet and nutrition: water, fruit and vegetable, and fiber increase; physical activity and function: physical activity-non-work 30 min, moderate activity
Miller et al. 2015 [27]	Body composition: body weight; diet and nutrition: intake of fruits, meat, fish, poultry, nuts, and seeds; physical activity and function: step counts; psychological health outcomes: self-efficacy, goal difficulty, and satisfaction with physical fitness, social support, and problem orientation (positive orientation); work-related or organizational outcomes: employee presenteeism
Audrey et al. 2015 [28]	Physical activity and function: daily minutes of moderate-to-vigorous physical activity (MVPA) and overall physical activity
Solenhill et al. 2016 [29]	Diet and nutrition: days of eating breakfast and sugar intake; physical activity and function: days of physical activity per week
Gregoski et al. 2016 [30]	Body composition: weight; work-related or organizational outcomes: attendance rates
Chandrasiri et al. 2016 [31]	Diet and nutrition: consumption of fresh fruits and vegetables; physical activity and function: physical inactivity
Hendriksen et al. 2016 [32]	Clinical health outcomes: SBP; diet and nutrition: fruit and vegetable consumption; physical activity and function: MVPA and sedentary behavior
Jamal et al. 2016 [33]	Clinical health outcomes: quality of life (WHOQOLBREF); body composition: weight, BMI, waist circumference, hip circumference; psychological health outcomes: perceived social support from friend (MDPSS), self-efficacy in weight management (WEL); diet and nutrition: intake of carbohydrates; physical activity: level of physical activity
Balk-Moller et al. 2017 [34]	Body composition: body weight, body fat percentage, and waist circumference
Maylor et al. 2018 [35]	Clinical health outcomes: SBP; and body composition: waist circumference and fat-free mass; physical activity and function: total steps
Viester et al. 2018 [36]	Body composition: body weight, BMI, and waist circumference; diet and nutrition: sugar-sweetened beverages; physical activity and function: MVPA (SQUASH)
Ing et al. 2018 [37]	Clinical health outcomes: SBP; body composition: weight; physical activity and function: the 6 min walk test (6MWT) and exercise frequency (physical activity questionnaire)
Kouwenhoven-Pasmooij et al. 2018 [38]	Clinical health outcomes: mental component score of SF-36; body composition: body weight and BMI; productivity and physical activity
Oftedal et al. 2019 [39]	Diet and nutrition: Australian Recommended Food Score (ARFS); physical activity and function, and the proportion of participants meeting physical activity guidelines
Fang et al. 2019 [40]	Clinical health outcomes: total cholesterol, triglyceride, LDL-C, HDL-C, WHOQOL-BREF (physical health, psychological health, social relationships, environment, overall QOL); body composition: weight, BMI, waist circumference, body fat percentage, number of MS risk factors; physical activity and function: flexibility, muscular strength, and endurance, cardiorespiratory endurance; work-related or organizational outcomes: work control, interpersonal relationship at work, global job satisfaction; psychological health outcomes: occupational work stress
Edman et al. 2019 [41]	Clinical health outcomes: self-reported symptoms (GI symptoms, headaches or migraines, fatigue, hemoglobin A1c, and blood pressure medication); body composition: weight; physical activity and function: aerobic and strengthening exercises (sessions/week); psychological health outcomes: perceived stress
Haufe et al. 2019 [42]	Clinical health outcomes: metabolic syndrome Z score, SBP, triglycerides, fasting glucose concentration, mental component score of SF-36; body composition: waist circumference, body weight, body fat percentage; physical activity and function: exercise capacity (peak power output): psychological health outcomes: anxiety and depression severity (HADS); work-related or organizational outcomes: Work Ability Index score (WAI); diet and nutrition: dietary quality scores
Stein et al. 2019 [43]	Physical activity and function: level of physical activity
Bonn et al. 2019 [44]	Physical activity: daily exercise activity time
Sareban et al. 2020 [45]	Clinical health outcomes: HbA1c, LDL-cholesterol; physical activity and function: commuting habits
Linnan et al. 2020 [46]	Diet and nutrition: dietary intake: fruits and vegetables (times/day), sugar-sweetened beverages (times/day), fast food/eating out (times/day), eating habits score (times/day); physical activity and function: level of physical activity, muscle-strengthening activities (times/week); sleep habits and quality: hours per night
Piao et al. 2020 [47]	Physical activity and function: the level of physical activity and change in Self-Report Habit Index (SRHI)
Reich et al. 2020 [48]	Clinical health outcomes: SBP, mental component score of SF-36; physical activity and function: exercise capacity (Pmax), walking (km), and cycling (km)
Haufe et al. 2021 [49]	Clinical health outcomes: alanine aminotransferase (ALT), aspartate aminotransferase (AST): alkaline phosphatase (AP), gamma-glutamyl transferase (gGT), fibrosis scores (APRI score); body composition: weight; physical activity and function: maximum power output during incremental exercise testing
Neshteruk et al. 2021 [50]	Physical activity and function: level of physical activity
Mamede et al. 2021 [51]	Physical activity and function: daily steps
Noori et al. 2021 [52]	Diet and nutrition: dietary quality scores; physical activity and function; psychological health outcomes: stress management, spiritual growth, interpersonal relations, health responsibility, total HPLP-II score
Stephenson et al. 2021 [53]	Physical activity and function: sedentary behavior
Garcia Perez de Sevilla et al. 2021 [54]	Clinical health outcomes health-related quality of life (SF-36); diet and nutrition: dietary quality scores; physical activity and function: daily sitting time (GPAQ)
Kong et al. 2022 [55]	Body composition: BMI, hip circumference, waist-to-height ratio; diet and nutrition: frequency of sweetened beverage, frequency of fruit intake, and frequency of vegetable intake; physical activity and function: daily steps
Shiri et al. 2022 [56]	Body composition: BMI; psychological health outcomes: the risk of depressive symptoms; sleep habits and quality: hours per night; work-related or organizational outcomes: work ability
Bayerle et al. 2022 [57]	Clinical health outcomes: SBP; body composition: body weight (kg), BMI (kg/m^2^), fat mass (kg), body fat (%), and relative exercise capacity Physical activity and function: total PA (MET × h/week) and everyday activity (MET × h/week); Psychological health outcomes: depression severity (HADS-D depression subscale); work-related or organizational outcomes: Work Ability Index (WAI)
Edwardson et al. 2022 [58]	Clinical health outcomes: biochemical; physical activity and function: daily sitting time; psychological health outcomes: Perceived Stress Scale (PSS)
Silva et al. 2022 [59]	Clinical health outcomes: WHOQOL-BREF (physical health domain, psychological health domain, environmental health domain); body composition: waist circumference, hip circumference, waist-to-height ratio (WHtR); physical activity and function: moderate-to-vigorous physical activity (MVPA)
Brinkmann et al. 2023 [60]	Clinical health outcomes: HbA1c, total cholesterol, LDL, triglycerides, SBP, DBP; body composition: body weight, BMI, waist circumference; physical activity and function: exercise capacity

**Table 5:** The success rate of the interventions in improving outcomes.

Outcome	No. of Studies Reporting Improvement	No. of Studies Reporting the Outcome	Success Rate (% Reporting at Least Some Improvement)
Clinical Health Outcomes	15	27	55.6%
Body Composition	16	29	55.2%
Diet and Nutrition	12	19	63.2%
PA and Function	26	32	81.3%
Sleep Habits and Quality	2	7	28.6%
Stress and Coping	4	5	80%
Depression and Anxiety	3	7	42.9%
Self-Efficacy	1	3	33.3%
Work-Related	7	16	43.8%

## Data Availability

The original contributions presented in the study are included in the article; further inquiries can be directed to the corresponding author.

## Supplementary Materials

The following supporting information can be downloaded at: https://www.mdpi.com/article/10.3390/healthcare13111292/s1.

## Abbreviations

The following abbreviations are used in this manuscript:

PAWHI	Physical activity-led workplace health interventions
PA	Physical activity
