# Supplementary material for: Effectiveness of Physical Activity-Led Workplace Health Promotion Interventions: A Systematic Review
Source: Healthcare (Basel). 2025 May 29;13(11):1292. doi: 10.3390/healthcare13111292 (PMC12154417; doi:10.3390/healthcare13111292)
Supplement: Supplementary file 1 [file healthcare-13-01292-s001.zip › Supplementary Material S1.pdf]

Table S1. Research Objectives and Questions

	Primary	Secondary
Objectives	1. Comprehensively evaluate the effectiveness of Physical Activity-led Workplace Health Interventions (PAWHI) in enhancing employee health and promoting healthy behaviors.<br>2. Analyze the success rates of physical activity interventions and other supplementary measures in improving employee health, offering scientific evidence and practical guidance for workplace health interventions.	1. Provide evidence-based recommendations for businesses and health management professionals to optimize the design and implementation of workplace health interventions.<br>2. Explore factors affecting intervention effectiveness, such as work environment and employee demographics, to achieve more comprehensive and sustainable health outcomes.
Questions	1. What specific impacts do Physical Activity-led health interventions have on employee health and organizational productivity?<br>2. How effective are different supplementary interventions combined with physical activity in improving various dimensions of employee health (e.g., physical, mental, nutritional)?	1. What are the economic cost-effectiveness and long-term return on investment of the current intervention programs?<br>2. How to balance the universality and personalization of interventions to meet diverse employee needs and enhance effectiveness?<br>3. What is the long-term effectiveness of workplace health interventions in improving employee health and organizational benefits?
