# Supplementary material for: Effectiveness of Physical Activity-Led Workplace Health Promotion Interventions: A Systematic Review
Source: Healthcare (Basel). 2025 May 29;13(11):1292. doi: 10.3390/healthcare13111292 (PMC12154417; doi:10.3390/healthcare13111292)
Supplement: Supplementary file 1 [file healthcare-13-01292-s001.zip › Supplementary Material S2.pdf]

The rating criteria of the Cochrane Risk of Bias tool

Category	Rating Standard
Random sequence generation (selection bias)	Low risk : The researchers described how to generate random sequences. For example: Use a random number table; Use a computer random number generator; Toss a coin Sealed cards or envelopes; "Throwing color son" Draw lots and so on.
	Unclear risk : There is insufficient information to determine it as high-risk or low-risk.
	High risk : The researchers described the methods for generating incorrect random sequences. Such as odd and even numbers or dates of birth; Admission date (or day of the week) Inpatient number or outpatient number. Or directly classify the subjects using non-random classification methods, such as: the doctor's judgment; The patient's performance Laboratory indicators The patient's requirements, etc.
Allocation concealment (selection bias)	Low risk : Neither the subjects nor the researchers could predict the allocation outcome: central randomization; Random sequence medicine boxes with the same appearance; Opaque, sealed envelopes with random sequences, etc.
	Unclear risk : There is insufficient information to determine the risk level.
	High risk : The subjects and researchers may be able to predict the allocation results, such as: open random allocation; There is no appropriate guarantee for the distribution of envelopes (such as not sealed, transparent, and not a random sequence); Alternating or cyclic enrollment; Date of birth Medical record number Any other explicit non-hidden programs.
Blinding of participants and personnel	Low risk : There was no blinding or incomplete blinding, but the review author (that is, you) determined that the outcome was unlikely to be affected by the absence of blinding; The subjects and

(performance bias)	the main researchers were blinded, and it was unlikely to break the blinding.
	Unclear risk : There is insufficient information to determine the risk level. Or not mentioned
	High risk : Although blinding or incomplete blinding was used, the outcome might be affected by the absence of blinding. Blinding was set for the subjects and the researchers responsible for recruitment, but it was possible to break the blinding, and the outcome might be affected by the absence of blinding methods.
Blinding of outcome assessment (detection bias)	Low risk : The outcomes were not blinded, but the review authors determined that the outcomes were unlikely to be affected by the absence of blinding. It ensures a blind evaluation of the outcome and is unlikely to be unblinded.
	Unclear risk : There is insufficient information to determine the risk level. Or it was not mentioned.
	High risk : The outcomes were not evaluated by blinding, but the review authors determined that the outcomes might be affected by the lack of blinding. Blinded evaluation of the outcome was conducted, but it might have been unblinded, and the measurement of the outcome might be affected by the absence of blinding.
Incomplete outcome data (attrition bias)	Low risk : There are no missing data in the outcome. The reason for the absence of outcome measures is unlikely to be related to the truth value of the outcome. The missing outcome measures were balanced among groups and for similar reasons. The absence of outcome measures is not sufficient to affect the effect of the intervention; The missing data was filled in by appropriate methods.
	Unclear risk : The information on follow-up or exclusion in the report is insufficient to determine the risk level. Or not mentioned.
	High risk : The reason for the absence of outcome measures may be related to the truth value of the outcome, and the number or cause of the absence is inconsistent among groups. The absence of outcome indicators is sufficient to affect the effect of the intervention; When a large number of interventions violate random allocation, the "as-treated" strategy should be applied for analysis; The missing data was filled with inappropriate methods.
Selective	Low risk : The research protocol is available and all pre-stated

reporting （reporting bias）	outcomes have been reported. The research plan is unavailable, but the published report contains all the expected results.
	Unclear risk：It is determined to be of the above two grades without sufficient information.
	High risk：One or more primary outcome measures were not previously stated; The review shows that one or more of the primary outcome measures of concern to the researchers were incompletely reported and could not be included in the analysis. The research report did not report the expected primary outcome.
Other bias	Low risk：There are no obvious other biases.
	Unclear risk：There is not sufficient information to evaluate whether there is a significant risk of bias. There is no sufficient reason or evidence to suggest that the existing problems will introduce bias.
	High risk：There are potential biases related to specific research designs; There is fraud; There are other problems.
